# Dynamic Changes of Soil Microbial Communities During the Afforestation of *Pinus Armandii* in a Karst Region of Southwest China

**DOI:** 10.1007/s00248-024-02345-8

**Published:** 2024-01-24

**Authors:** Bin He, Qing Li, Shun Zou, Xiaolong Bai, Wangjun Li, Yang Chen

**Affiliations:** 1 College of Ecological Engineering, Guizhou University of Engineering Science, Bijie City, 551700 Guizhou Province China; 2 Guizhou Province Key Laboratory of Ecological Protection and Restoration of Typical Plateau Wetlands, Bijie City, 551700 Guizhou Province China

**Keywords:** Bacterial Community, Fungal Community, *Pinus armandii.* Plantation, Stand age, Soil Properties, Karst Region

## Abstract

Clarifying the response of soil microbial communities to vegetation restoration is essential to comprehend biogeochemical processes and ensure the long-term viability of forest development. To assess the variations in soil microbial communities throughout the growth of *Pinus armandii* plantations in the karst region, we utilized the “space instead of time” approach and selected four *P. armandii* stands with ages ranging from 10 to 47 years, along with a grassland control. The microbial community structure was determined by conducting Illumina sequencing of the 16 S rRNA gene and the ITS gene, respectively. The results demonstrated that afforestation with *P. armandii* significantly influenced soil microbial communities, as indicated by notable differences in bacterial and fungal composition and diversity between the plantations and the control. However, soil microbe diversity did not display significant variation across stand ages. Moreover, the bacterial community exhibited higher responsiveness to age gradients compared to the fungal community. Soil physicochemical factors play a critical role in elucidating microbial diversity and community composition variations during restoration processes. TN, AN, TP, AP, SOC, AK, and pH were the most significant influencing factors for the composition of bacterial community, while TC, SOC, pH, and TC_a_ were the most significant influencing factors for the composition of fungal community. Our findings indicate substantial changes in soil bacterial and fungal communities across successive stages of development. Additionally, the changes in dominant bacteria and fungi characteristics across the age gradient were primarily attributed to variations in the prevailing soil conditions and chemical factors.

## Introduction

Globally, there are about 22 million km^2^ of karst area, accounting for 15% of the earth’s surface area [1]. As one of the three continuous karst distribution centers in the world [2], southwest China is characterized by strong and typical karst development, and extremely fragile ecosystem. Due to the prominent human-earth conflicts, coupled with irrational socio-economic activities irrationally [3], many serious ecological and environmental problems, especially rocky desertification, have occurred in this region. Conventionally, vegetation restoration has been recognized as an effective approach for enhancing degraded ecosystems and mitigating the spread of rocky deserts. Afforestation is a widely practiced technique for restoring degraded ecosystems [4]. Since the mid-1970s, the Chinese government has initiated several ecological restoration programs in this region, such as the Green for Grain program and the Natural Forest Protection Project. Significant progress has been achieved in afforestation in the Karst ecosystems of southwest China. Numerous studies have demonstrated that afforestation has substantial impacts on aboveground and underground ecosystems, resulting in ecological benefits [5], alteration of soil properties and microbial communities [6, 7], along with their interrelationships. Nevertheless, compared with soil physicochemical properties, limited information is available regarding how soil microbial assemblages respond to changes in vegetation characteristics during the establishment of secondary forests in the region.

Soil microorganisms are vital components of terrestrial ecosystems, playing a vital role in mediating the breakdown of organic materials, nutrient cycling, and energy flow [8]. They facilitate potential feedback along the continuum of soil-plant-atmosphere interactions, thereby contributing to ecosystem functioning [9]. Changes in the composition and diversity of microbial communities in various ecosystem processes can offer valuable insights into soil fertility status, serving as indicators of soil quality and disturbance intensity [10, 11]. Moreover, soil microorganism diversity and community composition are closely linked to soil ecosystem multi-functions [12]. Afforestation has a multifaceted impact on soil microbial communities, with regulation occurring simultaneously through various biotic and abiotic factors [13]. Previous research has demonstrated that abiotic factors, such as the availability of soil organic matter, pH, soil temperature, and water content, can influentially shape soil microbial communities [14]. Moreover, an imbalanced soil C:N:P stoichiometry can result in limited availability of nitrogen and phosphorus in the soil, consequently significantly impacting the composition and diversity of the soil microbial community [15]. Additionally, biotic factors, including the age of afforestation, tree species, community diversity, and functional traits of plants, possess the capability to directly or indirectly affect soil microbes via alterations in the nutrient cycle at the regional level [16, 17]. Specifically, the age of afforestation is predicted to lead to changes in available nutrient pools and energy flow in forest soils, thus affecting microbial communities. However, despite indications from previous studies suggesting that the age of afforestation is likely to influence the microbial community [18], the specific patterns of change in the microbial community remain uncertain. Some studies have observed an increase in microbial biomass with each successive year of planting [19], while others have found a decrease or no significant difference [20]. Similarly, the diversity and richness of microbial communities have been observed to either increase, decrease, or remain unchanged with planting age [21, 22]. The lack of consistency in these findings highlights the need for further research to understand the specific impacts of planting age on soil microbial communities. Such information is crucial for predicting the responses of soil microorganisms to future environmental changes and for better understanding successional dynamics mechanisms in afforested areas [23].

*Pinus armandii* is a distinctive five-needled pine species in China with a broad natural distribution range spanning an altitude gradient of 800 to 3,500 m. Due to its rapid growth, superior material quality, robust cold resistance, adaptability to arid and infertile soils, as well as its ability to thrive in limestone cracks, *P. armandii* is the primary tree species cultivated in the national key ecological construction project in southwest China. Previous studies have predominantly investigated the influence of *P. armandii* plantation on soil physicochemical properties, community structure, plant characteristics, and ecosystem function. However, limited attention has been given to the impact of these plantings on soil microbial communities, especially in fragile ecological environments. This information is essential for the future implementation of *P. armandii* in efforts to combat rocky desertification and restore degraded karst ecosystems.

Based on existing research and theory indicating the profound influence of afforestation on microbial communities, we formulated two hypotheses. Firstly, afforestation can cause changes in soil properties and microbial community structure, which will ultimately affect ecosystem function (biodiversity maintenance, material circulation, and energy flow, etc.). Considering the distinct adaptation strategies of bacteria and fungi to soil chemistry and resource availability [24], we hypothesized that the diversity of soil bacteria and fungi would demonstrate different dynamics following afforestation (Hypothesis I). Furthermore, it was anticipated that the relative abundances of dominant bacterial and fungal groups would be influenced by the age of the stand (Hypothesis II). To test these hypotheses, we conducted an analysis of soil chemical properties at four different time points in *P. armandii* plantations, comparing them to a control grassland. Concurrently, 16 S rRNA and ITS high-throughput sequencing technologies were employed to investigate the bacterial and fungal communities in the soil. The objectives of this study were as follows: (i) to assess changes in soil characteristics, microbial diversity, and composition within *P. armandii* plantations across different planting years; (ii) to explore the correlation between bacterial and fungal diversity, composition, and soil physicochemical properties; and (iii) to identify the primary factors driving variations in the composition and diversity of soil microbial communities throughout the restoration period.

## Materials and Methods

### Site Region

The study was conducted in Bijie City (26°21’N-27°46’N, 103°36’E-106°43’E), located in the northwest of Guizhou Province, China. This area is situated in an inclined zone that connects the eastern Yunnan Plateau to the central Guizhou mountains, characterized by complex geological structures, including alternating folds and faults. Sedimentary rocks constitute the majority of the exposed rocks in this area. The average altitude is 1600 m, and the relative elevation difference is large. This leads to a noticeable vertical climate change. The mean annual temperature in the area falls within the range of 10 to 15 °C, accompanied by average annual precipitation ranging from 849 to 1399 mm. The area experiences annual sunshine hours ranging from 1096 to 1769 h, and a frost-free period lasting 245 to 290 days. The predominant soil types in the area include yellow-brown soil, lime soil, and stony soil. The vegetation in this area is primarily composed of mid-subtropical evergreen broad-leaved forests, which are dominated by tree species such as *Quercus senescens*, *Cyclobalanopsis glauca*, and *Lithocarpus hancei*. Additionally, there are also mountain evergreen coniferous forests and mixed coniferous broad-leaved forests, with dominant tree species including *Pinus massoniana*, *P. armandi*, *Pinus yunnanensis*, and *Keteleeria evelyniana* [25].

### Experimental Design and Soil Sampling

Experimental sites were selected based on the distribution of *P. armandii* in the study area using the “space-for-time substitution” method. These sites included grassland (D00) and artificial forests of *P. armandii* aged 10, 16, 22, and 47 years, which were abbreviated as P10, P16, P22, and P47, respectively. Each of the five stand age classes was replicated three times, representing 15 stands. The sites shared similar geographical features and soil types. In August 2021, three 20 m x 20 m plots were randomly assigned to each stand for subsequent investigation and sampling. To ensure representative sampling, replicated plots of the same stand age were separated by at least 100 m, whereas the distances between different stand age classes generally exceeded 2 km. Table 1 provides the basic information for the plantations at different stand ages.

**Table 1:** Characteristics of *P. armandii.* plantation with different stand ages

Stand age	Coverage of the canopy (%)	Average diameter at breast height (cm)	Average height (m)	Stand density (N·hm^− 1^)
10	70 ± 5	10.22 ± 0.15	7.35 ± 0.07	3480 ± 25.23
16	74 ± 7	12.00 ± 0.22	9.29 ± 0.10	2538 ± 18.73
22	79 ± 6	15.71 ± 0.26	12.67 ± 0.16	2240 ± 11.02
47	85 ± 10	34.91 ± 0.93	22.83 ± 0.46	666 ± 5.78

Note: The data are means ± standard error of three replicates

After clearing away the litter layer and other debris, soil cores were gathered for each plot using a soil auger with a 5 cm diameter at a depth of 0–20 cm. To ensure representative soil samples, the soil cores were collected in an “S” shape pattern. A total of five soil cores were collected for each plot. Subsequently, the collected soil cores were combined to form a composite sample for each individual plot. To remove roots, litter, stones, and other debris, all soil samples were efficiently filtered using a 2 mm mesh. The composite soil sample was then divided into two sub-samples for subsequent analyses. One of the sub-samples was immediately packed into centrifuge tubes and promptly frozen in liquid nitrogen. These tubes were then stored in a dry ice box for transportation to the laboratory and were kept at a temperature of -80 °C to facilitate DNA extraction. The other sub-sample was packed into a sealed bag and brought back to the laboratory for the determination of soil physicochemical properties. This sample was air-dried in a dry and cool environment.

### Soil Physicochemical Properties

Soil pH, Total carbon (TC), Soil organic carbon (SOC), Total nitrogen (TN), available nitrogen (AN), Total phosphorus (TP), available phosphorus (AP), Total potassium (TK), available potassium (AK), and Total calcium (TCa) were measured. All of these chemical determination methods were used in accordance with the Bao approach [26].

### DNA Extraction and PCR Amplification

Total genomic DNA was extracted from the soil sample using the CTAB/SDS method according to the manufacturer’s instructions. The extracted DNA was evaluated for concentration and purity using 1% agarose gel electrophoresis. The final DNA concentrations were adjusted to 1 ng/µL using sterile water in order to prepare the sequencing libraries. For bacterial 16 S rRNA gene amplification, the V4 region was targeted using the PCR with the primers 515 F (50-GTGCCAGCMGCCGCGGTAA-30) and 806R (50-GGACTACHVGGGTWTCTAAT-30). For fungal rRNA gene amplification, the ITS1-5 F region was targeted using the primers ITS5-1737 F (50-GGAAGTAAAAGTCGTAACAAGG-30) and ITS2-2043R (50-GCTGCGTTCTTCATCGAT GC-30). Each PCR reaction included an amplification mixture consisting of 0.2 µM of each primer, 10 ng of target DNA, and 15 µL of Phusion® High-Fidelity PCR Master Mix (New England Biolabs). The PCR amplification was conducted using the following procedure: an initial denaturation step at 98℃ for 1 min, followed by 30 cycles of denaturation at 98℃ for 10 s, annealing at 50℃ for 30 s, extension at 72℃ for 30 s, and a final extension step of 5 min at 72℃. DNA was detected using 2% agarose gel, following the combination of PCR products with 1X loading buffer containing SYBR green. The NEBNext® Ultra™ II DNA Library Prep Kit (Cat No. E7645) was utilized for the production of the sequencing libraries. The quality and quantity of the libraries were assessed using the Qubit@ 2.0 Fluorometer from Thermo Scientific and Agilent Bioanalyzer 2100 system. Following this, the sequencing process was conducted on an Illumina NovaSeq platform at Novogene Bioinformatics Technology Co., Ltd. (Beijing, China), which generated 250 bp paired-end reads.

### Sequence Data Processing

Using QIIME2 software (Version QIIME2-202006), the sequences underwent quality filtering and chimera eradication. Prior to the removal of primer sequences and barcodes, raw sequence data were initially attributed to individual samples based on distinct barcodes. FLASH software (Version 1.2.11) was employed to merge the paired-end reads. For quality filtering of the raw tags, the Fastp software (version 0.20.0) was employed, while chimera checking and subsequent removal were performed using Vsearch (version 2.15.0). Identification of ITS sequences was executed through the Unite database, while 16 S rRNA sequences were determined using the Silva database. Using DADA2 in QIIME2 software (Version QIIME2-202006), the remaining sequences were clustered into operational taxonomic units (ASVs) with an abundance of more than 5. Subsequently, multiple sequence alignment was conducted in QIIME2 software to study the phylogenetic relationships of each sequence. The complete sequences have been submitted to the Sequence Read Archive (SRA) database of the National Center for Biotechnology Information (NCBI), with accession numbers PRJNA1063200. Alpha-diversity metrics, such as the number of observed OTUs, Chao1 richness estimator, and Shannon’s diversity index, were computed for each sample using QIIME2 software. Additionally, beta diversity, which assesses the complexity of community composition between samples, was determined based on weighted distances in QIIME2 software.

### Statistical Analyses

The normality assumption of the variables was assessed using the Kolmogorov-Smirnov test, which confirmed that all variables met the assumption of normality necessary for further variance analysis. The LSD and ANOVA methods were employed to determine variations in the relative abundance of the primary microbial phyla, microbial alpha diversity, and soil physicochemical parameters. Statistical significance was set at *P* < 0.05. The microbial community structures in the stands were evaluated using Principal Coordinates Analysis (PCoA). To identify differentially represented microbial taxa among different stand ages in the microbial community, the linear discriminant analysis effect size (LEfSe) method was applied. This analysis was carried out using the LEfSe tool. To retain meaningful taxa, rare taxa with a relative abundance below 0.0005 were excluded from the analysis. Taxonomic significance was determined based on an LDA score greater than 4 and a *P*-value less than 0.05. To identify soil factors that significantly influenced bacterial and fungal communities, redundancy analysis (RDA) was conducted with Monte Carlo permutations (999 iterations) to examine the relationships. The relationships between soil properties, microbial communities, and microbial diversity indices were assessed using the Spearman correlation coefficient. R software (version 4.0.0) was used to conduct all analyses. The models were conducted by the “innerplot” function of the package “plspm” in R (https://www.R-project.org/.Sinsabaugh).

## Results

### Soil Properties

Substantial variations in soil chemical properties were evident across different stand stages (Table 2). When compared to the grassland, afforestation resulted in varying trends in TC and SOC concentrations. The lowest concentrations were observed at the 16-year stage, while the highest concentrations were recorded at the 47-year stage. On the other hand, the concentrations of TN, AN, TP, AP, and AK consistently increased with stand age, showing significant increments ranging from 28.57 to 333.61%, 14.29–385.71%, 32–340%, 26.67–126.67%, and 50–275%, respectively. The concentrations of TK and TCa first increased and then decreased, peaking at the 22-year and 16-year stages, respectively. There was a consistent decrease in soil pH along the stand age gradient, dropping from 5.97 to 4.45. Additionally, the C:N and N:P ratios exhibited an uneven successional pattern, while the C:P ratio displayed a decrease followed by an increase.

**Table 2:** Soil properties of *P. armandii.* plantations with different stand ages

Samples	Grassland	10-y	16-y	22-y	47-y
TC (g·kg^− 1^)	42.47 ± 1.98d	62.85 ± 1.47b	25.68 ± 1.69e	57.50 ± 4.07c	68.74 ± 1.0a
SOC (g·kg^− 1^)	34.08 ± 2.56d	59.83 ± 0.34b	23.62 ± 1.60e	54.97 ± 3.57c	65.09 ± 0.90a
TN (g·kg^− 1^)	1.19 ± 0.02d	1.53 ± 0.29 cd	2.00 ± 0.09c	4.26 ± 0.35b	5.16 ± 0.37a
AN (g·kg^− 1^)	0.14 ± 0.03d	0.16 ± 0.02d	0.29 ± 0.02c	0.51 ± 0.03b	0.68 ± 0.05a
TP (g·kg^− 1^)	0.25 ± 0.00d	0.33 ± 0.09d	0.61 ± 0.13c	0.86 ± 0.11b	1.10 ± 0.11a
AP (g·kg^− 1^)	0.15 ± 0.01c	0.19 ± 0.03c	0.26 ± 0.04b	0.30 ± 0.02ab	0.34 ± 0.02a
TK (g·kg^− 1^)	3.55 ± 0.08c	3.93 ± 0.28c	6.47 ± 0.45b	7.81 ± 0.37a	6.52 ± 0.20b
AK (g·kg^− 1^)	0.04 ± 0.00c	0.06 ± 0.01c	0.11 ± 0.02b	0.12 ± 0.01b	0.15 ± 0.01a
TC_a_ (g·kg^− 1^)	0.18 ± 0.01d	0.25 ± 0.02d	1.84 ± 0.19a	1.32 ± 0.05b	0.95 ± 0.16c
pH	5.97 ± 0.09a	5.94 ± 0.14a	4.58 ± 0.19b	4.51 ± 0.07b	4.45 ± 0.05b
C:N	28.64 ± 1.68b	34.16 ± 1.05a	11.48 ± 0.17d	12.34 ± 0.22d	15.47 ± 0.05c
C:P	136.17 ± 12.01a	134.06 ± 4.72a	46.87 ± 1.60c	49.57 ± 2.79c	62.47 ± 0.80b
N:P	4.75 ± 0.15b	6.68 ± 0.22a	3.77 ± 0.40c	4.85 ± 0.18b	4.13 ± 0.38c

Note: 10-y, 16-y, 22-y and 47-y indicate *Pinus armandii.* plantations with stand ages of 10, 16, 22 and 47 years, respectively. TC, total soil carbon; SOC, soil organic carbon; TN, total nitrogen; AN, available nitrogen; TP, total phosphorous; AP, available phosphorous; TK, total potassium; AK, available potassium; TCa, total calcium; C:N, organic carbon to total nitrogen ratio; C:P, organic carbon to total phosphorus ratio; N:P, nitrogen to phosphorus ratio. Values are means ± standard error (*n* = 3). Different lowercase letters indicate significant differences (*P* < 0.05) among different stand ages based on a one-way ANOVA followed by an LSD test

### Soil Microbial Diversity

**Fig. 1:**
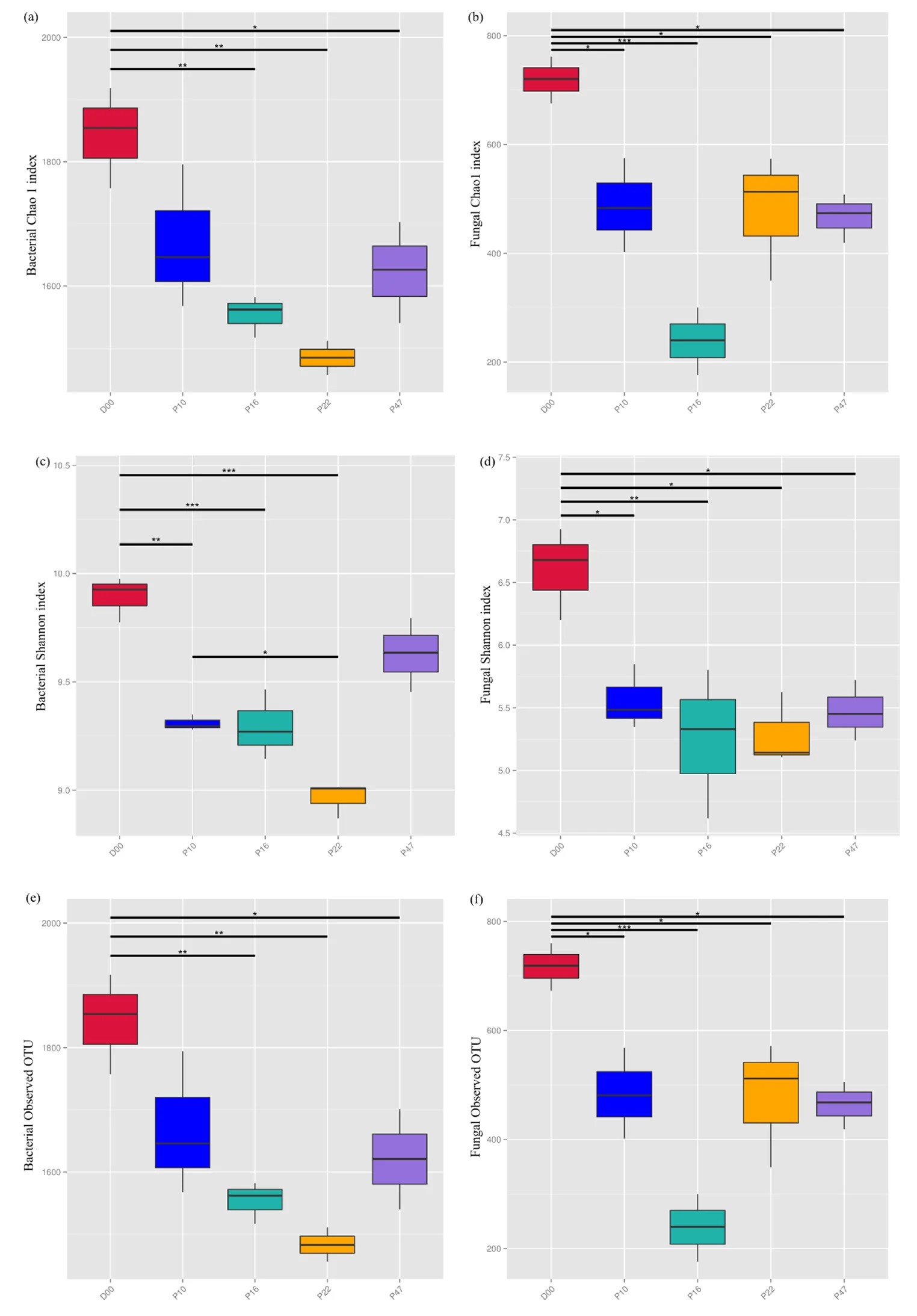
Diversity indices of soil bacterial and fungal communities at *P. armandii.* plantations with different stand ages. D00, P10, P16, P22 and Y47 indicate grassland and *P. armandii.* plantations with stand ages of 10, 16, 22, and 47 years, respectively

Afforestation had a significant impact on the α-diversity indexes of soil bacteria and fungi. The grassland soil harbored the highest richness and diversity of bacteria and fungi, as indicated by the number of OTUs, Chao l, and Shannon index (*P < 0.05*, Fig. 1). The average number of bacterial OTUs at different stand ages was 1842, 1669, 1554, 1483, and 1620, respectively. The α-diversity of soil bacteria initially decreased and then increased over time, reaching their lowest values at 22 years (Fig. 1a, c, e). The Shannon index of soil bacteria at 10 years was much higher than that at 22 years (*P < 0.05*). Regarding soil fungi, the average number of fungal OTUs was 717, 484, 239, 477, and 464 for different stand ages. The measurements of soil fungal diversity initially decreased but eventually reached a balanced trend in the later stages of succession, with their lowest point observed at 16 years (Fig. 1b, d, f).

**Fig. 2:**
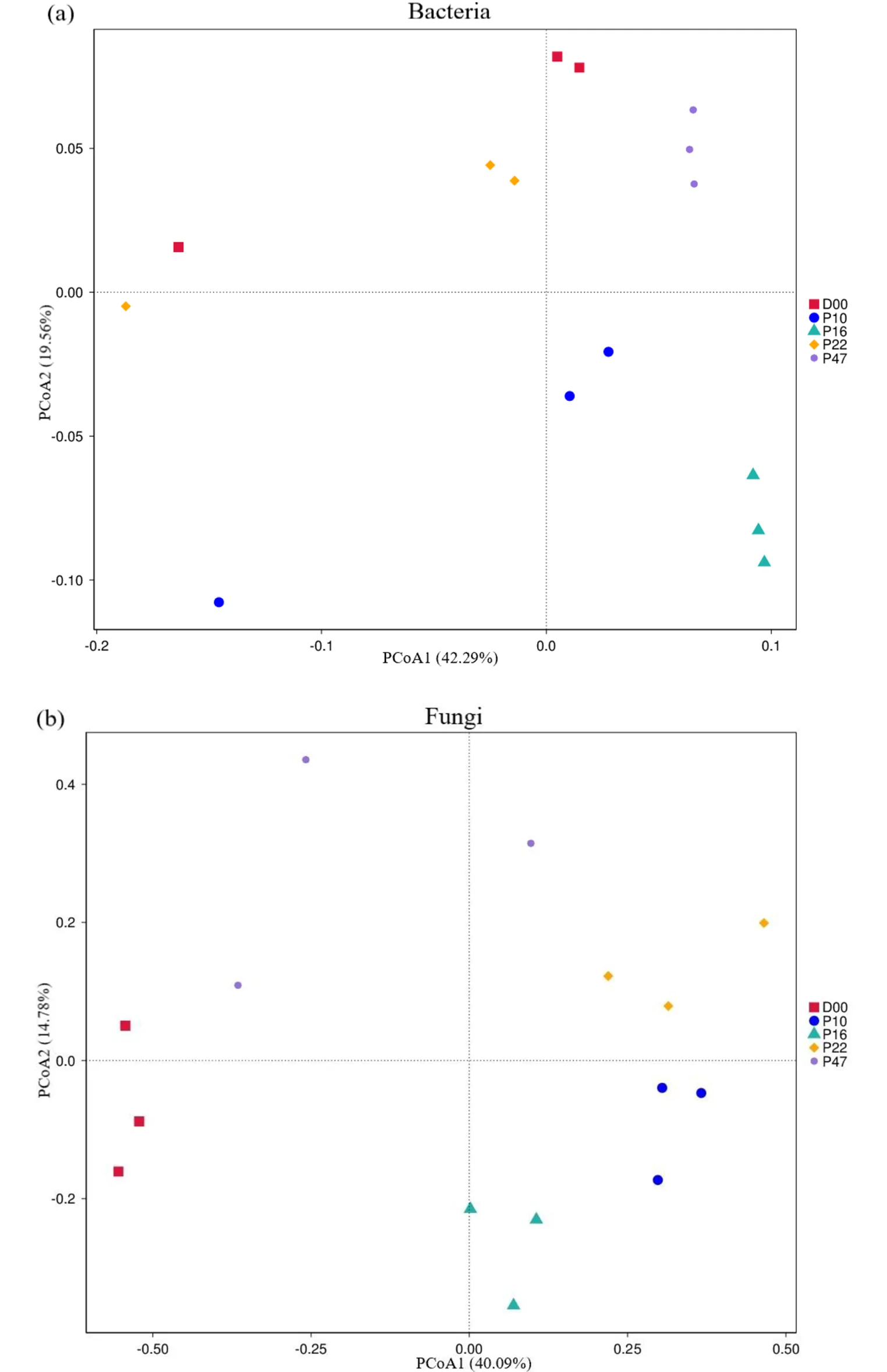
Principal coordinate analysis (PCoA) based on weighted Bray-Curtis for the bacterial (**a**) and fungal communities (**b**) at *P. armandii.* plantations with different stand ages. D00, grassland; P10, 10-y plantation; P16, 16-y plantation; P22, 22-y plantation; P47, 47-y plantation

The PCoA indicated variations in both bacterial and fungal communities across different stand ages (Fig. 2). For bacterial communities, the first and second principal components explained 42.29% and 19.56% of the total variation, respectively (Fig. 2a). The corresponding values for fungal communities were 40.09% and 14.78% (Fig. 2b).

### Soil Microbial Community Composition

The 16 S rRNA and ITS primer sets were utilized to acquire a large number of high-quality sequences from the soil samples, resulting in 897,647 bacterial sequences and 1,017,037 fungal sequences. It was observed that the number of fungal sequences obtained per sample ranged from 52,083 to 52,083 with a mean of 67,802. On the other hand, bacterial sequences exhibited a wider range from 53,436 to 66,159 per sample with a mean of 59,843. In subsequent analysis, the bacterial dataset was reduced to 11,496 sequences, while the fungal dataset was reduced to 3,399 sequences.

**Fig. 3:**
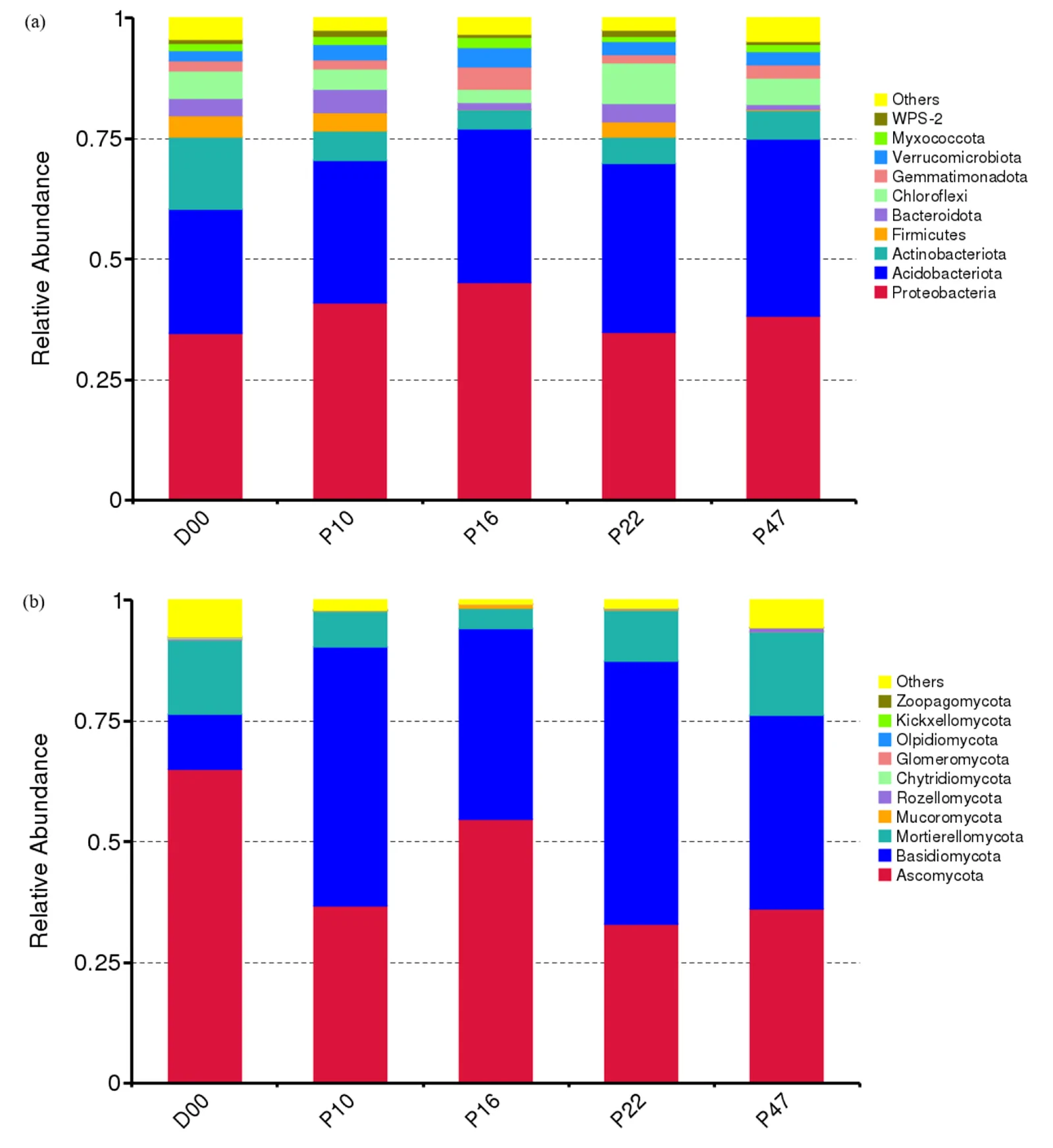
Relative abundance of dominant bacteria (**a**) and fungi (**b**) at the phylum level at *P. armandii.* plantations with different stand ages. The relative abundance level after the top 10 were defined as “Others”

Throughout the five plantation ages, a total of 37 bacterial phyla were identified in the community. The top ten prevalent phyla were Proteobacteria (38.81%), Acidobacteria (31.76%), Actinobacteria (7.32%), Chloroflexi (5.36%), Verrucomicrobiota (2.95%), Bacteroidota (2.92%), Gemmatimonadota (2.61%), Firmicutes (2.36%), Myxococcota (1.53%), and WPS-2 (0.95%) (Fig. 3a). Together, these ten phyla accounted for over 96% of the bacterial abundance. Notably, the relative abundance of different bacteria varied as the stand age increased. Actinobacteriota initially declined and subsequently increased, while Verrucomicrobiota exhibited the opposite pattern. Acidobacteriota showed a gradual increase over time. Other phyla of bacteria showed fluctuating trends during different stages of the plantation process.

A total of 13 fungal phyla were identified in the fungi community. The dominant taxa at the phylum level, based on taxonomic classification, were Ascomycota (45.10%), Basidiomycota (39.84%), Mortierellomycota (10.97%), Mucoromycota (0.27%), and Rozellomycota (0.21%) (Fig. 3b). These five phyla represented over 96% of the total fungal abundance. As the succession progressed, Mortierellomycota and Rozellomycota initially dropped and subsequently rose, but Mucoromycota exhibited the opposite tendency. Ascomycota and Basidiomycota varied across different stages of the plantation.

**Fig. 4:**
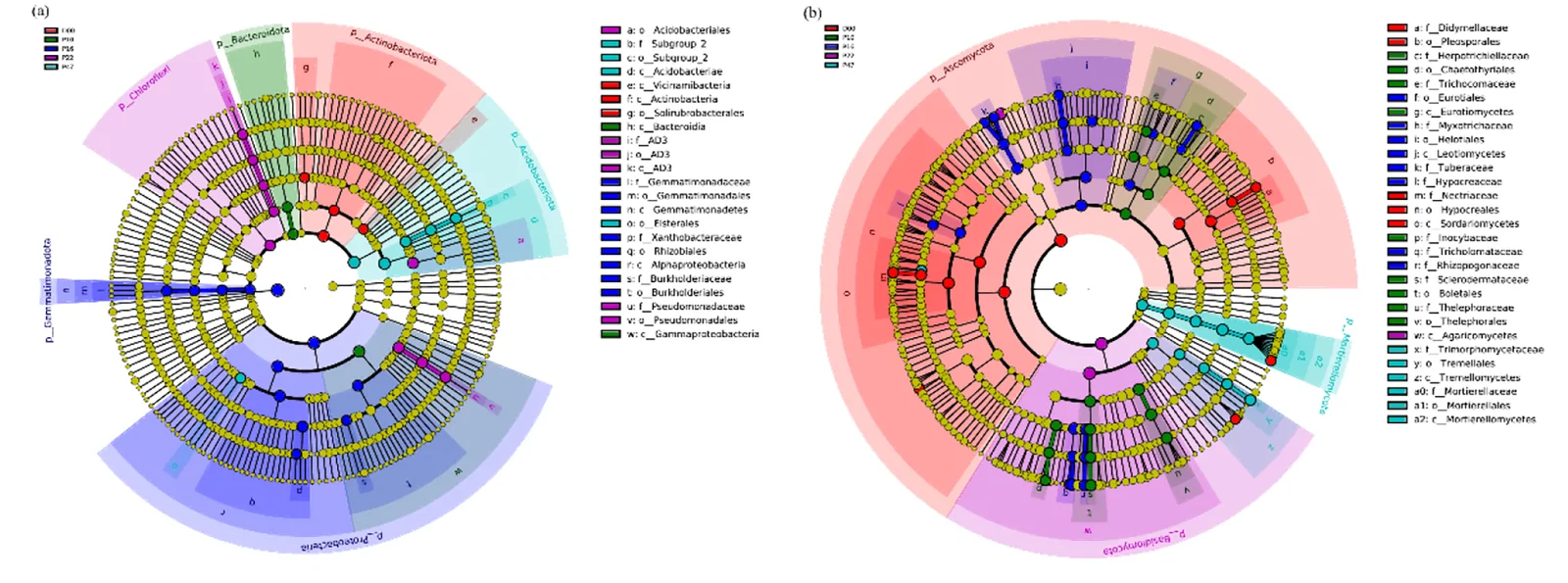
Cladogram demonstrates the distinctions between the enrichment groups for bacteria (**a**) and fungi (**b**). Colored dots show taxa with significant differences in abundance across different plantation stages, and Cladogram circles depict phylogenetic taxa from phylum to genus. For bacteria and fungi, only LDA scores higher than 4 were displayed. D00, grassland; P10, 10-y plantation; P16, 16-y plantation; P22, 22-y plantation; P47, 47-y plantation

The LEfSe analysis revealed significant changes in both bacterial and fungal communities in response to habitat change. Specifically, numerous bacterial and fungal groups exhibited significant enrichment at different stages of the plantation, ranging from phylum to genus levels (Fig. 4). In terms of bacteria, it was observed that the majority of taxa were enriched in the 16-year forest. For instance, the relative abundance of Gemmatimonadota (c_Gemmatimonadetes, o_Gemmatimonadales, f_Gemmatimonadaceae) and Proteobacteria (c_Alphaproteobacteria, o_Rhizobiales, f_Xanthobacteraceae, o_Burkholderiales, f_Burkholderiaceae) prevailed in the 16-year forest (Fig. 4a). Conversely, aside from the Basidiomycota (c_Agaricomycetes), which exhibited a pronounced increase in the 22-year forest, the majority of fungal taxa were enriched in the grassland habitat (Fig. 4b).

### Relationships Between Microbial Communities and Soil Properties

The association between soil microbial community diversity and soil properties was assessed through Spearman’s correlation analysis (Table 3). The results revealed a significant negative correlation between TK and the numbers of OTUs and the Chao1 Index of soil bacteria (*P < 0.05*). However, the remaining soil characteristics did not exhibit a significant relationship with the alpha diversity of bacteria. Additionally, no significant correlation was found between fungal diversity and soil physicochemical characteristics.

**Table 3:** Correlation analysis between soil and soil microbial diversity

Soil factors	Bacteria				Fungi		
	Chao1	Observed_otus	Shannon		Chao1	Observed_otus	Shannon
TC	0.462	0.462	0.573		0.448	0.420	0.210
SOC	0.448	0.448	0.531		0.566	0.552	0.336
TN	-0.252	-0.252	0.182		0.182	0.224	-0.119
AN	-0.266	-0.266	0.189		0.112	0.154	-0.112
TP	-0.189	-0.189	0.245		0.161	0.203	-0.119
AP	-0.322	-0.322	0.224		-0.007	0.042	-0.399
TK	-0.685*	-0.685*	-0.441		0.000	0.056	-0.273
AK	-0.063	-0.063	0.406		0.042	0.070	-0.203
TC_a_	-0.573	-0.573	-0.392		-0.552	-0.510	-0.336
pH	0.025	0.025	-0.235		-0.238	-0.259	0.284
C:N	0.469	0.469	0.308		0.573	0.538	0.245
C:P	0.455	0.455	0.294		0.503	0.462	0.301
N:P	0.084	0.084	-0.315		0.490	0.469	0.224

Note: “*” indicate significance at *P* < 0.05

**Fig. 5:**
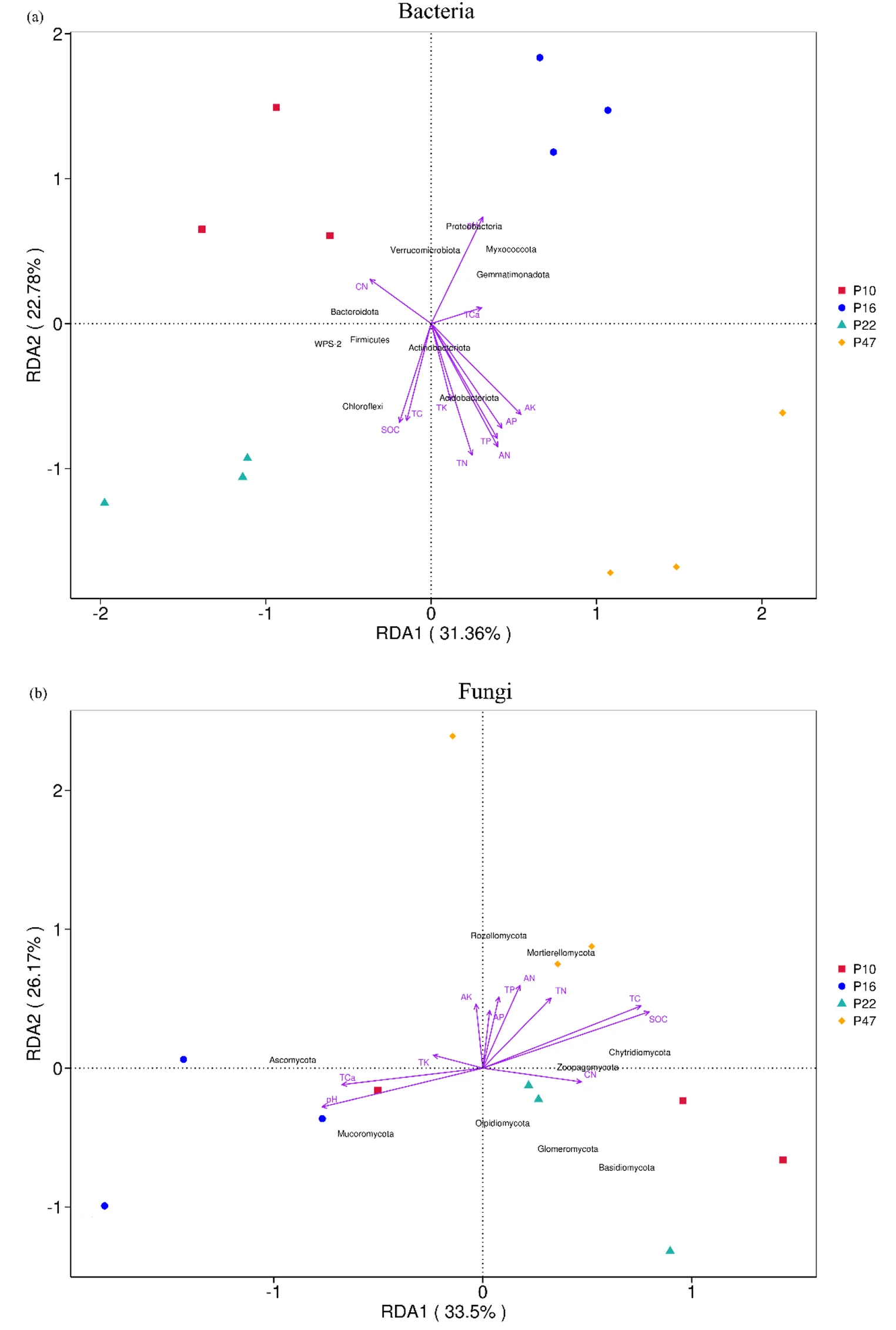
Redundancy analysis (RDA) identified the relationship between dominant bacteria (**a**) and fungi (**b**) at the phylum level and soil properties. D00, grassland; P10, 10-y plantation; P16, 16-y plantation; P22, 22-y plantation; P47, 47-y plantation

**Fig. 6:**
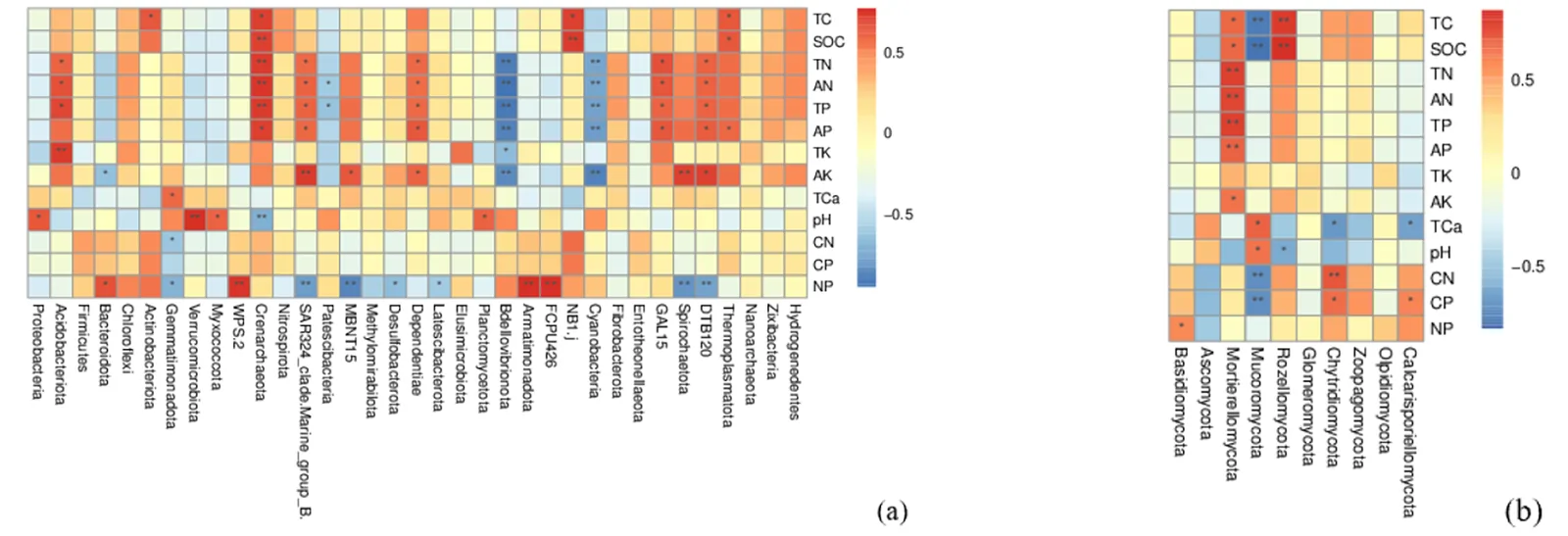
Spearman’s rank correlation coefficients between the bacteria (**a**) and fungi (**b**) at the phylum level and soil properties “*” and “**” indicate significant at *P* < 0.05 and *P* < 0.01, respectively

The soil’s physicochemical characteristics exhibited a significant influence on the composition of both bacterial and fungal communities (Fig. 5). The RDA analysis revealed that the first two canonical axes accounted for 54.14% of the relationship between soil factors and bacteria at the phylum level (Fig. 5a). Our findings indicated that TN, AN, TP, AP, SOC, AK, and pH were the key factors influencing the composition of the bacterial community (*P < 0.01*, *P < 0.05*, Table 4). Spearman correlation analysis was conducted to further investigate the relationship between soil properties and the composition of soil bacterial communities at the phylum level (Fig. 6a). The contents of TN, AN, TP, and AP exhibited significant positive correlations with the relative abundances of DTB120, GAL15, Dependentiae, SAR324 clade.Marine_group_B., and Crenarchaeota (*P < 0.01*, *P < 0.05*), while exhibiting a strong negative correlation with the relative abundance of Cyanobacteria and Bdellovibrionota (*P < 0.01*, *P < 0.05*). AK contents displayed a robust positive correlation with the relative abundances of DTB120, Spirochaetota, Dependentiae, MBNT15, and SAR324 clade.Marine_group_B., while showing a strong negative correlation with the relative abundances of Cyanobacteria, Bacteroidota, and Bdellovibrionota (*P < 0.01*, *P < 0.05*). pH exhibited significant positive correlations with the relative abundances of Planctomycetota, Myxococcota, Verrucomicrobiota, and Proteobacteria, but was significantly negatively correlated with the relative abundance of Crenarchaeota (*P < 0.01*, *P < 0.05*). SOC showed significant positive correlations with the relative abundances of Thermoplasmatota, NB1.j, and Crenarchaeota (*P < 0.01*, *P < 0.05*).

**Table 4:** Relationships of bacterial and fungal community compositions with soil factors identified by Envfit function tests

Soil factors	Bacteria			Fungi	
	R^2^	P		R^2^	*P*
TC	0.471	0.061		0.775	0.001**
SOC	0.502	0.039 *		0.800	0.001**
TN	0.886	0.001**		0.362	0.128
AN	0.886	0.001**		0.387	0.111
TP	0.788	0.001**		0.269	0.251
AP	0.704	0.009**		0.174	0.425
TK	0.297	0.218		0.065	0.748
AK	0.687	0.006**		0.214	0.355
TC_a_	0.106	0.588		0.469	0.045*
pH	0.639	0.011*		0.670	0.014*
C:N	0.230	0.318		0.233	0.308
C:P	0.228	0.321		0.239	0.301
N:P	0.463	0.067		0.396	0.096

Note: “*” and “**” indicate significance at *P* < 0.05 and *P* < 0.01, respectively

RDA analysis demonstrated that the relationship between soil factors and fungus at the phylum level was accounted for 59.67% across the first two canonical axes (Fig. 5b). The Envfit function test revealed that key factors influencing the fungal community structure were TC, SOC, pH, and TC_a_ (*P < 0.01*, *P < 0.05*, Table 4). Significant positive correlations were observed between the contents of TC and SOC and the relative abundance of Rozellomycota and Mortierellomycota, while a significant negative correlation was found with the abundance of Mucoromycota (*P < 0.01*, *P < 0.05*, Fig. 6b). pH exhibited a significant positive correlation with the relative abundances of Mucoromycota, but at the same time, it exhibited significant negative correlations with the relative abundance of Rozellomycota (*P < 0.01*, *P < 0.05*, Fig. 6b). The contents of TC_a_ showed a significant negative with the relative abundance of Calcarisporiellomycota and Chytridiomycota, but conversely, it exhibited a significant positive correlation with the relative abundance of Mucoromycota (*P < 0.01*, *P < 0.05*, Fig. 6b).

## Discussion

### Changes in Soil Microbial Community Diversity Following Afforestation

Plant succession depends heavily on the interactions between aboveground plants and belowground microorganisms [27]. Previous research has consistently demonstrated that as forests undergo development, changes in soil physicochemical properties, root biomass, and chemical composition lead to inevitable alterations in the diversity and composition of the soil microbial community [20]. Our study found that afforestation significantly influenced the diversity and richness of soil microbial communities. Notably, there were no significant differences observed in the composition and diversity of the communities between stands of varying ages (Fig. 1). However, in *P. armandii* plantations, there were various tendencies of changes in the diversity of bacteria and fungi with successive planting (Fig. 1). Soil pH has been identified as a major factor in determining the diversity of soil bacteria and fungi [28]. Our study did not find a statistically significant relationship between soil microbial diversity and pH (Table 3), leading us to assume that other factors may have influenced changes in the diversity of soil bacteria and fungi in the study area. Nevertheless, we cannot disregard the potential impact of pH on soil microbes, as generally low pH levels can inhibit metabolic activity and alter metabolic function [29]. Additionally, bacterial communities appear to be more influenced by the chronosequence and vegetation cover than fungal populations [30]. When undergoing successional changes, the richness of soil bacterial communities showed similarities to the species richness of overlying plants [31]. In our results, changes in soil bacterial diversity were similar to changes in the species richness of understory vegetation, which may help explain this relationship. While Waldrop et al. [32] argue that fungal diversity is not directly influenced by plant variety, plant communities indirectly affect fungal diversity through changes in litter production, which limits growth and reduces microbial biomass. Moreover, it was believed that distinct functional characteristics of plant species were related to the establishment of soil fungus communities [33].

In our study, the soil bacterial diversity initially decreased and then increased with the increasing age of *P. armandii* plantation (Fig. 1a, c, e), suggesting that soil conditions may become more favorable for bacterial growth and reproduction. It is generally considered that soil microbial biomass and activity are controlled by the quality and availability of soil carbon and nutrients. The ratio of carbon to nutrients can reflect the quality of the soil microbial growth environment to some extent [34]. Correlation analysis revealed a significant negative correlation between the number of OTUs and the Chao1 index of soil bacteria with soil TK (*P < 0.05*, Table 3), which supports the previous conclusion. The patterns of soil bacterial diversity change with age were in agreement with the findings of Wang et al. [35], who observed a gradual decline in the Chao1 and Shannon indices of soil bacteria from 6 to 25 years, followed by an increase from 25 to 49 years in a Chinese fir plantation. However, our results differ from the findings of Qu et al. [36], who reported a linear increase in bacterial diversity and richness in *Eucalyptus* plantations, and from the observations of Pan et al. [37], who found a linear decrease in the Shannon index with increasing age in a *P. massoniana* plantation. Moreover, Jiang et al. [38] discovered higher richness and diversity of the soil bacterial community in forestland compared to grassland, which contradicts our finding that the grassland exhibited the highest bacterial diversity indices (Fig. 1a, c, e). These inconsistencies highlight the need for further research to determine whether the diversity of soil bacteria changes during periods of secondary vegetation succession in similar patterns.

In contrast to bacteria, the diversity of the fungal community in the *P. armandii* plantation initially decreased and then tended to stabilize after 16 years of age (Fig. 1b, d, f). These findings imply that the fungal community attains a temporally stable state over time in response to external stimuli, specifically when succession has progressed to a specific stage [39]. This aligns with the research of Zhao et al. [40], who demonstrated that the OTUs of grassland soil fungi tend to stabilize during the later stages of Alpine grassland succession. Additionally, Knelman et al. [41] found that the dominance of a single plant type over time can influence the direction and homogenization of microbial communities. However, a contrasting viewpoint has been proposed, namely, that succession-related changes to the soil environment may make microbial communities malleable rather than static [42]. For instance, Zhu et al. [43] discovered a significant decrease in fungal community diversity indices after succession in a broad-leaved Korean pine forest. In a similar vein, Zhang et al. [44] reported an initial increase in fungal community diversity during the early stages of succession, followed by a decline after agricultural abandonment.

### Changes in Soil Microbial Community Composition After Afforestation

Our findings revealed successional patterns in the fungal and bacterial communities of the soil during the transition from grassland to afforestation (Fig. 3). Moreover, distinct dynamic patterns were observed within the prominent groups of bacterial and fungal phyla in the soil. Despite the prominent shift in vegetation from grassland to forest, the Proteobacteria phylum, which constitutes the most prevalent type of bacteria, maintained its dominance across different stand ages. However, a noticeable replacement occurred in the fungal community after afforestation. The dominant fungal phylum in the grassland, Ascomycota, was replaced by Basidiomycota in the *P. armandii* plantation. There is a possibility that variations in the succession of soil bacteria and fungi may be due to the diverse metabolic capabilities of the organisms and their ability to adapt to changes in environmental conditions and nutrient availability [24].

In this study, Proteobacteria and Acidobacteria were identified as the most dominant phyla at the bacterial level and had the highest impact on the variability of the microbial community across various habitat types (Fig. 3a). This finding aligns with previous studies that have also observed the prevalence of these bacterial groups in soil ecosystems [45]. Notably, after 16 years of plantation, the relative abundance of Proteobacteria decreased, while there was an increase in the relative abundance of Acidobacteria and Actinobacteria (Fig. 3a). This change in abundance indicated that the bacterial community has transitioned from R-strategy to K-strategy with the increasing age of *P. armandii*. Therefore, it can be inferred that oligotrophic bacteria may exert a suppressive effect on copiotrophic groups during the later stages of *P. armandii.* growth. However, it should be noted that many microorganisms within a given phylum do not strictly fit into either the copiotroph or oligotroph category due to the significant taxonomic variation present [46]. Although the precise definitions of oligotrophs and copiotrophs at the phylum level are still a subject of debate, it is widely acknowledged that microorganisms associated with these life strategies have unique physiological characteristics that enable predictions regarding their response to vegetation restoration [46].

Regardless of the succession stage, the fungal phyla Ascomycota, Basidiomycota, and Mortierellomycota exhibited dominance (Fig. 3b), which is in line with other studies [47]. Soil fungi can specialize in terms of their ecological requirements and their role in the ecosystem [48]. In the current study, there was a shift in the predominant fungal phylum from Ascomycetes in grassland to Basidiomycetes in the forest (Fig. 3b), indicating a transition from R- to K-strategy possibly due to habitat alterations. In forests, Basidiomycota fungi are prevalent because of the mycorrhizal associations formed by Agaricales and the decomposition of plant litter by other Basidiomycota fungi [49]. The Hypocreales and Sordariales were the two most prevalent orders among the Ascomycota phylum in our soils. Additionally, the majority of Sordariales are saprophytic and frequently found on decomposing plant material or dung [50]. The prevalence of Ascomycota in grassland soils can be attributed to the Sordariales’ association with animal dung, which is commonly present in the grassland areas of our study region. This finding aligns with Sun et al. [51], who observed that during the succession of the Loess Plateau, microbial communities at both the phylum and genus levels switched from R- to K-strategists. It is worth noting that the shift in the dominant fungal phylum in our soil corresponds to the fungal patterns observed in the Damma glacier forefield in central Switzerland during primary succession [49]. However, it is currently uncertain whether the transition from Ascomycota-dominant to Basidiomycota-dominant during primary and secondary succession is a universal pattern due to the use of a different method for analyzing the fungal community in the Damma glacier forefield compared to our present study.

The analysis of LEfSe provides valuable insights into the response of soil fungi and bacteria to changes in soil. The results indicate that Proteobacteria and Gemmatimonadota showed high enrichment in the 16-year plantation (Fig. 4a), suggesting their stable presence in this specific environment. Conversely, only the Basidiomycota exhibited significant enrichment in the 22-year plantation, while the major groups of fungi showed significant enrichment in the grassland (Fig. 4b). It is widely accepted that in a long-term stable community, characterized by minimal interference, a few highly competitive species will thrive and dominate the population [52]. Thus, the significant enrichment of Basidiomycetes in the 22-year plantation provides an additional explanation for the decrease in fungal diversity. Furthermore, the enrichment of both bacteria and fungi at the genus level aligns with the trends observed at the phylum level. Consequently, focusing research efforts on studying rare microorganisms at the genus level would produce more fruitful outcomes.

### Factors Influencing Soil Microbial Communities During Afforestation

The composition of the soil microbial population serves as an indicator of the impact of afforestation on the entire ecosystem, including its structure and functioning. Our research uncovered that afforestation significantly influenced the bacterial and fungal communities in the soil, as well as on soil physicochemical characteristics (Table 2; Figs. 2 and 3). The PCoA results revealed distinct dissimilarities among soil microbial communities across different stand ages (Fig. 2). Additionally, afforestation led to significant alterations in soil physicochemical properties in our study area (Table 2). These changes, including an increase in soil moisture, nutrient availability, and vegetation cover, indicated modifications in the soil habitat, thereby influencing the soil microbial communities (Fig. 3). Importantly, our findings suggest that soil bacterial communities exhibit higher sensitivity to changes in soil properties, particularly in terms of stability and diversity, compared to soil fungal communities. This sensitivity can be leveraged to enhance the availability of soil nutrients and labile carbon [53].

Studying the impact of soil parameters on soil microbial community composition during forest succession can enhance our understanding of how soil microbial communities change during forest succession [54]. The results of our study showed a significant impact of pH and SOC concentration on soil microbial populations in *P. armandii* plantations (Table 4; Fig. 5). Soil pH is considered the primary factor in shaping soil microbial communities [55]. The acidity of the soil, caused by root exudates during stand growth, might have contributed to these results. SOC concentration was also a key element in the transition of class-level community organization during the successional stages. It is worth noting that SOC has a limited impact on the diversity of soil bacterial community composition but significantly influences the growth of fungal communities (Fig. 5). Our findings align with previous research, which showed that fungi are more responsive to soil C incorporation into biomass compared to bacteria [56]. This can be attributed to the use of different carbon sources by bacteria and fungi. According to Liu et al. [57], the types of SOC become more complex and challenging to degrade during vegetation regeneration. Nevertheless, only a small number of fungi can release enzymes that facilitate the decomposition of complex organic C compounds like lignin. Currently, the microbial organisms capable of producing these enzymes are predominantly identified in the upper Basidiomycota group [58, 59].

The concentrations of TN, AN, TP, AP, and AK had the greatest impact on the composition of the bacterial community, as revealed by the RDA analysis (Fig. 5a) and Envfit function test (Table 4). This finding aligns with previous studies that have demonstrated the sensitivity of soil bacterial community composition to changes in soil characteristics, such as SOC, TN, and nitrate [34]. Specifically, SOC, TN, AN, TP, and AP exhibited positive correlations with the abundance of Acidobacteria and Actinobacteria, while demonstrating negative correlations with the abundance of Proteobacteria (Fig. 6a). Additionally, the N:P ratio demonstrated a significant negative correlation with DTB120, Spirochaetota, MBNT15, SAR324 clade.Marine_group_B., Latescibacterota, Desulfolbacterota, and Gemmatimonadota, but a strong positive correlation with FCPU426, Armatimonadota, WPS2, and Bacteroidota (Fig. 6a). The growth rate hypothesis [60] suggests that bacterial growth is primarily associated with an increasing demand for phosphorus as ribosomal RNA is synthesized. In previous studies, it was found that a close association between NO_3_^−^-N and the relative abundances of bacterial communities during the natural succession of abandoned fields on the loess plateau [61]. Likewise, our findings suggest that the availability of C, N, and P components is critical in determining the makeup of the soil bacterial community, with P and N likely acting as limiting factors in determining the composition of the soil bacterial community.

The main fungal phyla, primarily consisting of Ascomycota and Basidiomycota, exhibited weaker connections to soil physicochemical parameters. This suggests that the composition of fungal communities may not be a sensitive indicator of changing edaphic conditions resulting from ecosystem changes [62]. Interestingly, during succession, TC, SOC, and TCa concentrations showed significant associations with fungal groups (Table 4; Fig. 5b), which contradicted findings from other studies. This discrepancy may be explained by the reliance of soil fungi development on specific substrate types and their quality [63]. Kivlin et al. [64] discovered that the key process governing niche-driven fungal community assembly is environmental or abiotic filtering. Furthermore, Talbot et al. [65] demonstrated that plant traits and local environmental conditions explained just a small amount of fungal community variation relative to spatial distance in North America’s Pinaceae forestland. In contrast to the bacterial community, the population of soil fungi in the plant-soil system was more influenced by the C:N:P stoichiometric ratio (Fig. 6). The function of fungi was the primary cause of these differences. Ascomycetes and Basidiomycota, for example, possess the ability to efficiently degrade plant organic wastes, and the degradation of plant residues affects their abundance [66]. Consequently, the restoration of vegetation can promote the breakdown of plant wastes, enhancing C and N accumulation and conversion [67] and influencing the C:N:P stoichiometric ratio [68].

## Conclusion

The findings revealed that the cultivation of *P. armandii* considerably altered soil characteristics and microbial communities in the karst region of Southwest China. During the transition from grassland to forest, the diversity of bacteria initially decreased and then increased, whereas the diversity of fungi initially decreased and then stabilized in later succession stages. This indicated that bacteria and fungi followed distinct successional pathways. Furthermore, there was a noticeable shift in the composition of the microbial community over time. After 16 years of plantation, the relative abundance of Proteobacteria declined, while Acidobacteria and Actinobacteria increased. In the fungal community, there was a shift from Ascomycetes dominance in the grassland to Basidiomycetes dominance in the forest. These findings indicate that afforestation in this area led to a change from R-strategy to K-strategy dominant groups in the soil bacterial and fungal community composition. Moreover, soil physicochemical properties exerted a significant influence on the composition and diversity of microbial communities, particularly on soil bacterial dynamics. Specifically, TN, AN, TP, AP, SOC, AK, and pH were the most significant influencing factors for the composition of bacterial community. On the other hand, TC, SOC, pH, and TC_a_ were the most significant influencing factors for the composition of fungal community. In conclusion, this study provided valuable guidance for the restoration of *P. armandii* forests in the karst region of southwestern China.
